# A biologically-inspired multi-joint soft exosuit that can reduce the energy cost of loaded walking

**DOI:** 10.1186/s12984-016-0150-9

**Published:** 2016-05-12

**Authors:** Fausto A. Panizzolo, Ignacio Galiana, Alan T. Asbeck, Christopher Siviy, Kai Schmidt, Kenneth G. Holt, Conor J. Walsh

**Affiliations:** John A. Paulson School of Engineering and Applied Sciences, Harvard University, 29 Oxford Street, Cambridge, MA 02138 USA; Wyss Institute for Biologically Inspired Engineering at Harvard, 3 Blackfan Circle, Boston, MA 02115 USA; Department of Physical Therapy & Athletic Training, Boston University, Boston, MA 02215 USA

**Keywords:** Soft exosuit, Metabolic power, Loaded walking, Lower limb exoskeleton

## Abstract

**Background:**

Carrying load alters normal walking, imposes additional stress to the musculoskeletal system, and results in an increase in energy consumption and a consequent earlier onset of fatigue. This phenomenon is largely due to increased work requirements in lower extremity joints, in turn requiring higher muscle activation. The aim of this work was to assess the biomechanical and physiological effects of a multi-joint soft exosuit that applies assistive torques to the biological hip and ankle joints during loaded walking.

**Methods:**

The exosuit was evaluated under three conditions: powered (*EXO_ON*), unpowered (*EXO_OFF)* and unpowered removing the equivalent mass of the device (*EXO_OFF_EMR*). Seven participants walked on an instrumented split-belt treadmill and carried a load equivalent to 30 % their body mass. We assessed their metabolic cost of walking, kinetics, kinematics, and lower limb muscle activation using a portable gas analysis system, motion capture system, and surface electromyography.

**Results:**

Our results showed that the exosuit could deliver controlled forces to a wearer. Net metabolic power in the *EXO_ON* condition (7.5 ± 0.6 W kg^−1^) was 7.3 ± 5.0 % and 14.2 ± 6.1 % lower than in the *EXO_OFF_EMR* condition (7.9 ± 0.8 W kg^−1^; *p* = 0.027) and in the *EXO_OFF* condition (8.5 ± 0.9 W kg^−1^; *p* = 0.005), respectively. The exosuit also reduced the total joint positive biological work (sum of hip, knee and ankle) when comparing the *EXO_ON* condition (1.06 ± 0.16 J kg^−1^) with respect to the *EXO_OFF* condition (1.28 ± 0.26 J kg^−1^; *p* = 0.020) and to the *EXO_OFF_EMR* condition (1.22 ± 0.21 J kg^−1^; *p* = 0.007).

**Conclusions:**

The results of the present work demonstrate for the first time that a soft wearable robot can improve walking economy. These findings pave the way for future assistive devices that may enhance or restore gait in other applications.

**Electronic supplementary material:**

The online version of this article (doi:10.1186/s12984-016-0150-9) contains supplementary material, which is available to authorized users.

## Background

Carrying heavy loads alters the biomechanics of walking, leading to an increased metabolic burden. This negative consequence of load carriage has been reported in soldiers, first responders, and recreational athletes who are required to execute physically demanding tasks during walking [1, 2]. Several studies investigating the locomotion of these populations reported increased lower limb joint work [3, 4], which requires higher muscle activation to both sustain the load and stabilize the joints themselves [5]. Higher muscle activity is associated with an increased metabolic cost [5], leading to an earlier onset of fatigue and an overall reduction of performance [1, 2] while walking. Additionally, prolonged load carriage can result in an increased risk of injury, the most common of which are foot blisters, stress fractures, back strains, metatarsalgia (foot pain), rucksack palsy (shoulder traction injury) and knee pain [6]. Solutions that effectively reduce the burden associated with load carriage during walking are thus warranted.

Lower-limb exoskeletons have been proposed as a means to augment or assist human locomotion for many applications [7]. Some exoskeletons have been designed to make load carriage easier by providing a parallel load path to the ground [8–10], while others apply torques directly to the wearer’s joints [7, 11–14]. These systems are composed of rigid frames that allow the transmission of high forces and, although they represent remarkable achievements, their rigid nature presents a number of practical challenges toward the goal of assisting locomotion. The main challenges arise in aligning the exoskeleton and biological joints with each other [15] and reducing system mass and in particular distal mass as this can increase metabolic effort [16].

**Fig. 1:**
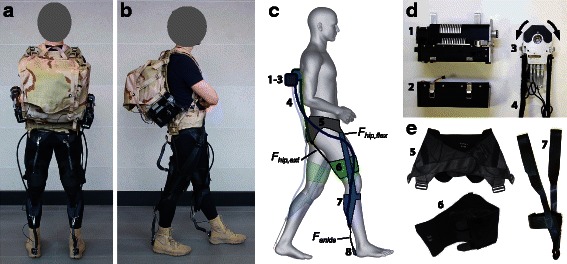
Soft exosuit components. **a** and **b** Back and side view of a participant wearing the soft exosuit. The two actuator units were mounted on an empty backpack and the exosuit was worn from the waist down. **c** Schematic drawing highlighting the two load paths of the soft exosuit, namely a monoarticular path assisting hip extension (*green*) and a multiarticular path assisting both hip flexion and ankle plantarflexion (*blu*e). Both load paths share the waist belt (grey). Numbers correspond to the actuation and suit components in (**d**) and (**e**). **d** Mechanics and electronic elements composing the actuator system. Motor (1), battery module (2) and multi-wrap pulley (3). **e** Textiles elements composing the soft exosuit. Waist belt (5), thigh brace (6) and calf strap (7)

As an alternative to rigid exoskeletons, we have developed a multi-joint soft exosuit [17–22] that uses textiles to provide a more compliant means to interface with the human body (Fig. 1a-b). Our exosuit is lightweight, with the majority of mass worn close to the wearer’s center of mass (Additional file 1: Table S1, which compares the weight with other autonomous exoskeletons), minimizing its impact on the energetics of gait [16]. The soft exosuit transmits moments around the biological joint axes through flexible cable-based transmissions and textiles that anchor to the body. Moreover, the exosuit minimally influences the wearer’s natural walking kinematics [17] and is active only when it detects walking. At all other times, the exosuit can be truly transparent when the cables are commanded to go slack. For this study, the exosuit assisted walking by generating assistive torques at the ankle and the hip, since they are the major power contributors to level-ground walking [23], via forces in two load paths (Fig. 1c), each actuated by a proximally-mounted actuation unit (Fig. 1d).

Our research group has demonstrated reductions in metabolic cost during load carriage with a tethered soft exosuit [20, 24]. One study [20], conducted with a lab-based, multi-joint tethered actuation platform (composed of a power supply, linear actuators and motor controllers mounted on a stationary platform next to a treadmill), reported reductions in the metabolic cost of walking for hip extension assistance (4.6 %) and for multi-joint assistance (14.6 %). Multi-joint assistance consisted of hip extension, ankle plantarflexion and hip flexion. Though promising, the tethered actuation platform limits the soft exosuit’s applicability to everyday walking.

Therefore, the aim of this work was to perform the first study with an autonomous (fully portable) multi-joint (assisting hip extension, ankle plantarflexion and hip flexion as in [20]) soft exosuit to evaluate if it could represent an effective solution to reduce the metabolic cost during loaded walking. We evaluated the performance of our soft exosuit on a group of load carriers walking with a load equivalent to 30 % their body weight under three conditions: with the device powered (*EXO_ON*), with the device unpowered (*EXO_OFF*) and with the device unpowered with equivalent mass removed (*EXO_OFF_EMR)*. The second condition (*EXO_OFF*) was evaluated to assess the penalty associated with carrying the additional mass represented by the device itself, an important consideration in the design of such systems. To obtain additional insights on the benefit of wearing the soft exosuit and to extend the knowledge on the biomechanical and physiological effects of this device, we evaluated metabolic cost, muscle activation and joint mechanics which have been shown to be relevant for regulating metabolic energy cost during gait [25].

## Methods

### Soft exosuit design and operation

**Fig. 2:**
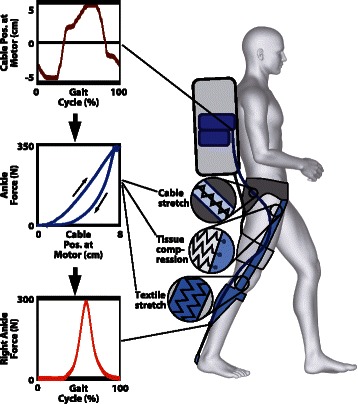
Suit-human series stiffness. This schematic illustrates the mapping between the cable position and the applied external force at the ankle [24]. The three panels on the left describe the effect of the suit-human series stiffness on force generation. The motor pulls on a Bowden cable thus regulating its position across the gait cycle (top panel). This position is transformed into a force on the wearer through the suit-human series stiffness, a non-linear relationship between the force measured at the ankle and the cable position of the motor (middle panel) that is due to the presence of series elastic elements. These elements include cable stretch, soft tissue compression and textile stretch. A force-based feedback loop in the control system ensures the application of a consistent force profile (bottom panel) accounting for the little variations applied by the suit-human series stiffness [24]. The description of the present schematic is relative to the multiarticular load path but an analogous behavior is present in the monoarticular load path

A lower extremity soft exosuit (Fig. 1a-c) and the associated actuation system (Fig. 1d) were fabricated. The exosuit and the actuation system were discussed in detail in [22]. Briefly, the exosuit consisted of a structured textile extending bilaterally from the waist to the feet and was composed of three principle components: a waist belt, bilateral thigh pieces and bilateral calf straps (Fig. 1e). Two actuation units (Fig. 1a-b) were mounted on a backpack and connected to the exosuit by Bowden cables. To operate, the actuation units retracted the inner cable of the Bowden cable assembly, delivering a controlled force to the wearer. The level of force transmitted to the wearer was monitored by load cells using force-based position control, as described in Additional file 1: Text S1 and in Additional file 1: Figure S1. Briefly, force-based position control imposes a predefined position trajectory as a function of the gait cycle to the motor acting on the Bowden cables to achieve a specific force profile. The cable position trajectory generates a force in the exosuit because the textile, the Bowden cables, and the human tissue underneath the exosuit are compliant. The combination of these factors, defined as the “suit-human series stiffness” (Fig. 2), is a mapping between the cable position and induced force. While the position control method generated consistent force profiles for fixed kinematics and suit conditions, an algorithm that monitors key force profile features iteratively adjusted assistive position profiles on a step-by-step basis to account for small variations in gait and drift of textile components. Similar iterative learning techniques to achieve desired exoskeleton torque patterns have been explored [26].

The load-transferring elements in the exosuit followed two distinct paths in each leg as described in Fig. 1c. One multiarticular load path extended from the waist, over the front of the thigh, down the side of the leg, passing approximately through the knee joint axis, and to the back of the calf. An additional supporting element attached at the front of the shin. A Bowden cable sheath was anchored to this load path at the back of the calf, with the inner cable extending to the back of the heel. A second monoarticular load path extended from the waist to the back of the hip, and down to the thigh. Along this load path, a Bowden cable sheath connected to the waist belt at the back of the hip, and the inner cable connected to the back of a thigh brace (Fig. 1).

Two actuation units (Fig. 1d) connected to the exosuit via the Bowden cables generated external forces along these two load paths. The multiarticular load path thus enabled the generation of both ankle plantarflexion and hip flexion torques, whereas the monoarticular load path generated hip extension torques.

The ankle and hip contribute together approximately 80 % of the positive mechanical power produced by the lower limb joints during walking [23, 27]. Power generation at the ankle occurs mainly as the body is transitioning from one leg to the other. The ankle on the trailing leg plantarflexes the foot, which propels the body upward and forward to reduce the impact on the leading leg that is accepting the weight of the body. Conversely, power at the hip is generated during extension when it accepts the body’s weight just after heel strike and during flexion when it helps the body to propel forward [27]. This second burst of power generation at the hip occurs as the leg begins to swing forward, which is approximately coincident with the ankle’s pushing off. Therefore, our bioinspired design enables actuation of both joints with a single load path. Details of how the force in the multiarticular load path contributes to hip flexion are explained further in Additional file 1: Text S1.

### Soft exosuit actuation and control

The actuation system we built for the exosuit enabled two motors to actuate all four of the load paths (two load paths per leg). Each of the two actuation units (Fig. 1d) consisted of: (1) a module with a geared motor, (2) a multi-wrap pulley connected to two Bowden cables, (3) electronics and (4) a battery module. One actuation unit controlled the bilateral multiarticular load paths, whereas the other actuation unit controlled the bilateral monoarticular load paths as previously described in [22]. In each case, when the motor turned clockwise, the load path on the left leg developed tension while the load path on the right leg was made slack so that no force could be generated. When the motor turned counterclockwise, the load path on the right leg developed tension and the left leg was made slack. Thus, this approach enabled the exosuit to become completely transparent along a particular load path when desired. The total mass of the multi-joint soft exosuit (including the soft exosuit and the two actuation units) was 6.6 kg.

### Participants

We recruited seven load carriers from the local community (age: 29.3 ± 6.2 yr; height: 1.80 ± 0.07 m; weight: 77.9 ± 8.3 kg). All participants reported no musculoskeletal injuries or diseases and provided written informed consent. The participants whose images appear in the manuscript have provided written consent for the publication of their images according to the policies of the Journal of NeuroEngineering and Rehabilitation. The study was approved by the Harvard Medical School Committee on Human Studies.

### Testing protocol

**Fig. 3:**
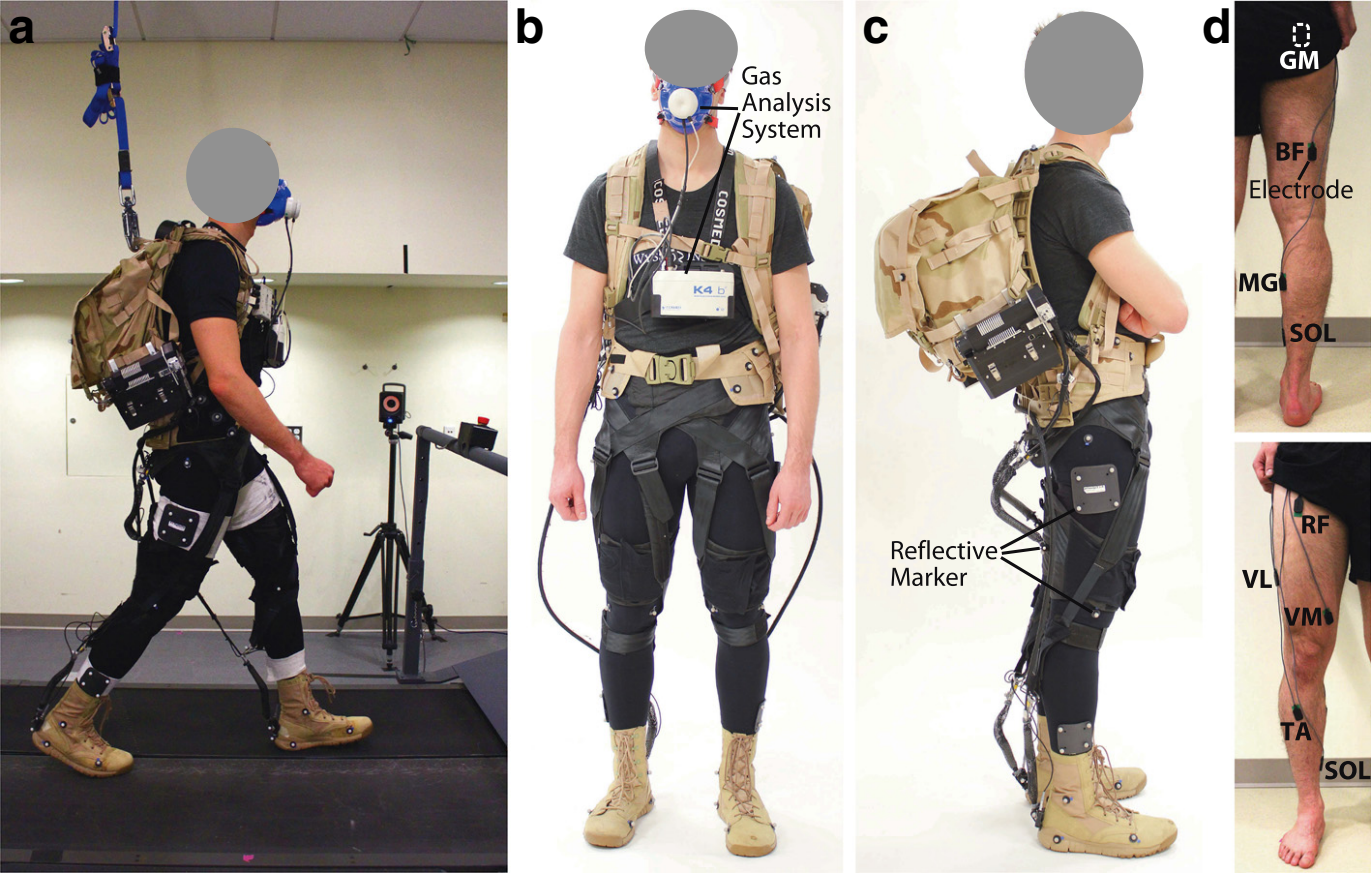
Experimental methods. **a** Data collection representing an instrumented participant carrying a loaded backpack and wearing the soft exosuit while walking on a split-belt treadmill (Bertec, Columbus, OH, USA). **b**-**c** An instrumented participant, front and side view. Metabolic cost is measured by means of portable gas analysis system (K4b^2^, Cosmed, Roma, Italy) and participant’s kinematics are measured by means of a 3D motion capture system (VICON, Oxford Metrics, UK; 120 Hz) tracking the position of 50 reflective markers placed on the participant. **d** Placement of surface electrodes (Delsys, Natick, MA, USA) on the lower limb muscles investigated, back and front view: rectus femoris (RF), vastus medialis (VM), vastus lateralis (VL), gluteus maximus (GM), biceps femoris (BF), soleus (SOL), medial gastrocnemius (MG) and tibialis anterior (TA)

Three different walking conditions were evaluated in the present study: *EXO_ON, EXO_OFF* and *EXO_OFF_EMR*. Participants walked on an instrumented split-belt treadmill measuring three-dimensional ground reaction forces (Bertec, Columbus, OH, USA; 2160 Hz) at a constant speed of 1.5 m s^−1^ while carrying a load equivalent to 30 % of their body mass (Fig. 3a) with the actuation units powered (*EXO_ON*), and unpowered (*EXO_OFF* and *EXO_OFF_EMR*). In the *EXO_OFF_EMR* condition, used as baseline comparison, the equivalent weight of the multi-joint soft exosuit (6.6 kg) was removed from the load in the backpack, further details on this procedure are provided in Additional file 1: Text S1. Participants wore the same boots (Nike SFB) in all the conditions, as this footwear is representative of that worn by load carriers. These boots were worn because they had an integrated attachment on the heel (Fig. 1a) to fix the inner cable of the Bowden cable assembly, and have weight comparable to that of walking shoes (450 g for size 9). Participants walked for six minutes in the *EXO_OFF_EMR* and in the *EXO_OFF* condition and for nine minutes in the *EXO_ON* condition. The first three minutes of the *EXO_ON* condition allowed the device’s controller to ramp up forces to the desired level (detailed description in Additional file 1: Text S1). The three walking conditions were randomized for each participant to avoid any learning or adaptation effects. Participants underwent a training session at least two days prior to the data collection sessions to allow them to become familiar with the exosuit and its operation before evaluations. In this session, participants completed two bouts of walking with the exosuit powered and unpowered, for the same duration and with the same modality of the *EXO_ON* and the *EXO_OFF* conditions.

### Metabolic cost

Metabolic cost during walking was assessed by indirect calorimetry using a portable gas analysis system (K4b^2^, Cosmed, Roma, Italy) (Fig. 3b). Carbon dioxide and oxygen rate were averaged across the last two minutes of each condition used to calculate metabolic power using a modified Brockway equation [28]. Net metabolic power for each testing condition was obtained by subtracting the metabolic power obtained during a standing trial performed at the beginning of each session from the metabolic power calculated during the walking conditions. Net metabolic power was normalized by the body mass of each participant.

### Joints kinematics and kinetics

Three-dimensional (3D) gait analysis was performed during treadmill walking. The marker set used for 3D motion capture (VICON, Oxford Metrics, UK; 120 Hz) was composed of 50 markers placed on specific anatomical bony landmarks (Fig. 3b-c). Single markers were placed on the left and right legs at the calcanei, heads of the first and fifth metatarsals, medial and lateral malleoli, medial and lateral knee condyles, greater trochanters, left and right anterior superior iliac spines, left and right iliac crests and at the midpoint between the iliac crest and the anterior superior iliac spine on the left and right side. Clusters of four markers were attached to the thighs and shanks of both legs. Eight additional markers were placed on the proximal and distal ends of each cable at the ankle and hip, used to calculate the assistive forces’ lines of action, along with their associated moment arms. All markers and force trajectories were filtered using a zero-lag 4^th^ order low pass Butterworth filter with a 5–9 Hz optimal cut-off frequency selected using a custom residual analysis algorithm (MATLAB, The MathWorks Inc., USA). Joint angles, net joint moments and powers were calculated in the sagittal plane using filtered markers and forces by means of an inverse kinematic and dynamic approach (Visual 3D, C-Motion, Rockville, MD, USA). Net joint moments and powers were then normalized by each participant’s body mass. An automatic gait event detection algorithm (Visual 3D, C-Motion, Rockville, MD, USA) was used to determine heel strikes that defined gait cycles. Ten strides per condition were used for generating mean kinematic and kinetic data for each participant, which were subsequently combined to calculate condition mean data.

### Muscle activity

During all trials, surface electromyography (EMG) signals from eight lower limb muscles were measured by means of a wired system (Delsys, Natick, MA, USA; 2160 Hz) simultaneously with the motion data measured by the VICON system. Muscles investigated were: rectus femoris (RF), vastus medialis (VM), vastus lateralis (VL), gluteus maximus (GM), biceps femoris (BF), soleus (SOL), medial gastrocnemius (MG) and tibialis anterior (TA) (Fig. 3d). EMG signals were band-pass filtered (4th order Butterworth, cut-off 20–450 Hz), rectified and low-pass filtered (4th order Butterworth, cut-off 6 Hz) to obtain a linear envelope. For each participant and for each muscle, the EMG linear envelope was normalized to the peak value (averaged across ten strides) recorded during the *EXO_OFF_EMR* condition. Ten strides per condition were used to compute mean muscle activation across each stride.

### Biological joint work

**Fig. 4:**
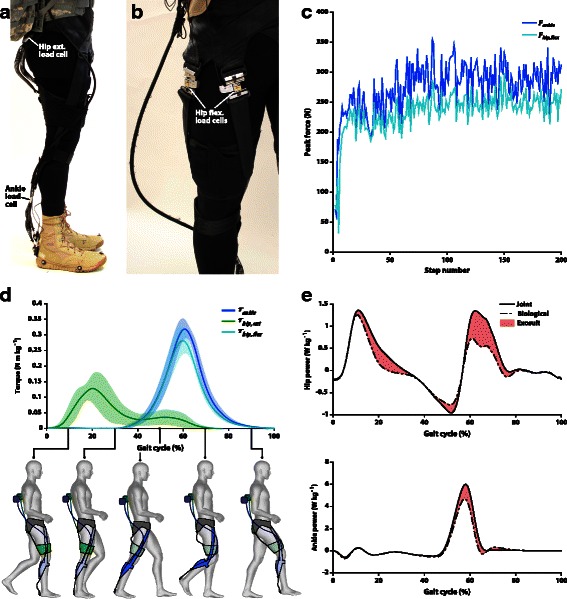
Assistance applied by the soft exosuit to the wearer. **a** Side view of the soft exosuit highlighting the load cells at the hip and at the ankle used to quantify the level of mechanical assistance provided by the soft exosuit. **b** Experimental setup highlighting the load cells inserted in the soft exosuit to assess contribution of the hip extension external force during the exosuit characterization experiment. **c** Peak force at the waist (light blue) and peak force at the ankle (blue) recorded during the exosuit characterization experiment. The waist peak force is the sum of the peak forces collected by the two load cells placed at the front of the thigh and the ankle peak force was collected directly by the load cell placed on the ankle. Data are relative to one representative participant. **d** Torque profiles at the ankle (blue) and at hip flexion (green) recorded during the testing sessions across all the participants involved in the study; estimated torque profile at the hip extension (light blue) calculated during the exosuit characterization experiment. The schematic drawing illustrates the assistance provided by the exosuit during the phases of the gait cycle. The multiarticular load path is displayed in blue and the monoarticular path is displayed in green. **e** Joint power (black) and biological joint power (dashed black) calculated for hip and ankle during the *EXO_ON* condition. The shaded area represents the power provided by the exosuit. Data are group means

To compute the biological components of net joint moment and power during the *EXO_ON* condition, the actuation units were synchronized to the Vicon system using a 5 V signal generated at the beginning of the motion capture data collection. Forces measured by load cells at the ankle and the hip during the *EXO_ON* condition (Fig. 4a) were segmented based on the heel strike times obtained by the automatic gait events detection algorithm. Ten strides were used to obtain an average force profile during the gait cycle. Ankle and hip extension moments generated by the exosuit during the *EXO_ON* condition (Fig. 4d) were calculated for each participant as the product of the force recorded by the corresponding (ankle or hip) load cell and the computed moment arms. Moment arms were defined as the perpendicular distance between the markers on the cable and the respective joint center. As described above, the multiarticular nature of the exosuit generated a hip flexion moment while assisting with ankle plantarflexion. To quantify this flexion moment, an exosuit characterization experiment was conducted on a separate testing day as described in Additional file 1: Text S1. Briefly, two additional load cells were placed at the point where the leg straps connect to the waist belt (Fig. 4b). To assess the force distribution in the multiarticular load path, the peak force collected by the ankle load cell was compared to the sum of the peak forces collected by the load cells at the waist belt (Fig. 4c). In this way it was possible to assess which percentage of the force applied at the ankle was also applied to the hip flexion through the multiarticular load path. This estimated peak force was multiplied by a constant moment arm to compute the assistive moment during hip flexion. The biological joint moments produced during the *EXO_ON* condition were then calculated by subtracting the moment generated by the exosuit at the ankle (or hip) from the net joint moment at the ankle (or hip) as per [29]. Biological moment was then multiplied by joint velocity to obtain biological power (Fig. 4e). Lastly, biological positive and negative joint work for the *EXO_ON* condition were calculated for the ankle and the hip joints of the right leg by integrating over time the positive and negative corresponding biological powers. For the *EXO_OFF_EMR* and the *EXO_OFF* conditions, positive and negative biological work were calculated by inverse dynamics.

### Statistical methods

Statistical analysis was conducted in SPSS (SPSS Inc., Statistics21, USA). One-way repeated measures analyses of variance (ANOVA) with three modes (*EXO_OFF_EMR*, *EXO_OFF* and *EXO_ON*) were used to verify the effect of the device on metabolic power and on the average muscle activation across the stride. The biomechanical variables of interest included: peak flexion and extension joint angles and moments, as well as absorbed and generated joint power (positive and negative area of the power trace curves) at the ankle, knee and hip. Additional one-way repeated measures ANOVA were conducted on positive and negative biological joint work and power (total and single joint). Bonferroni *post hoc* tests were performed to identify differences between conditions when a statistically significant main effect was identified by the ANOVA. The significance level was set at *p* < 0.05 for all analyses. Effect sizes were calculated using Cohen’s d method.

## Results

### Metabolic cost and muscle activity

**Fig. 5:**
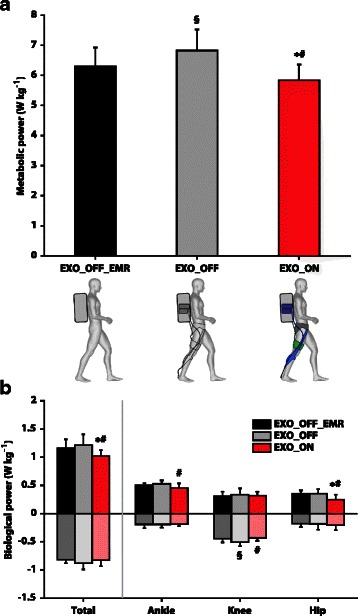
Metabolic power and biological power. **a** Metabolic power reported in the three conditions of testing: *EXO_OFF_EMR* (black), *EXO_OFF* (grey) and *EXO_ON* (red). **b** Biological negative and positive power across the lower limb joints and for each single joint reported in the three conditions of testing: *EXO_OFF_EMR* (black), *EXO_OFF* (grey) and *EXO_ON* (red). Data are means ± SD. * and § indicate significant difference (*p* < 0.05) with respect to the *EXO_OFF_EMR* condition, # indicates a significant difference (*p* < 0.05) with respect to the *EXO_OFF* condition

Average metabolic power during standing with a load equivalent to 30 % of each participant’s body mass was, on average, 1.6 ± 0.3 W kg^−1^. Net metabolic power was, on average, 7.9 ± 0.8 W kg^−1^, 7.5 ± 0.6 W kg^−1^, and 8.5 ± 0.9 W kg^−1^ during the *EXO_OFF_EMR*, the *EXO_ON*, and the *EXO_OFF* conditions, respectively. Net metabolic power for the *EXO_ON* condition was 7.3 ± 5.0 % (*p* = 0.027; ES = 0.57) and 14.2 ± 6.1 % (*p* = 0.005; ES = 1.31) lower than in the *EXO_OFF_EMR* and for the *EXO_OFF* conditions, respectively. This resulted in a significant metabolic power reduction during the *EXO_ON* condition of 35.2 ± 23.0 W (*p* = 0.020; ES = 0.63) and 74.8 ± 33.6 W (*p* = 0.003; ES = 1.36) with respect to the *EXO_OFF_EMR* and to the *EXO_OFF* condition, respectively (Fig. 5a). Furthermore, for every 1 J of exosuit positive mechanical work generated, participants saved, on average, 1.8 J of metabolic energy. Each participant’s metabolic power during the three test conditions is presented in Additional file 1: Table S2.

**Fig. 6:**
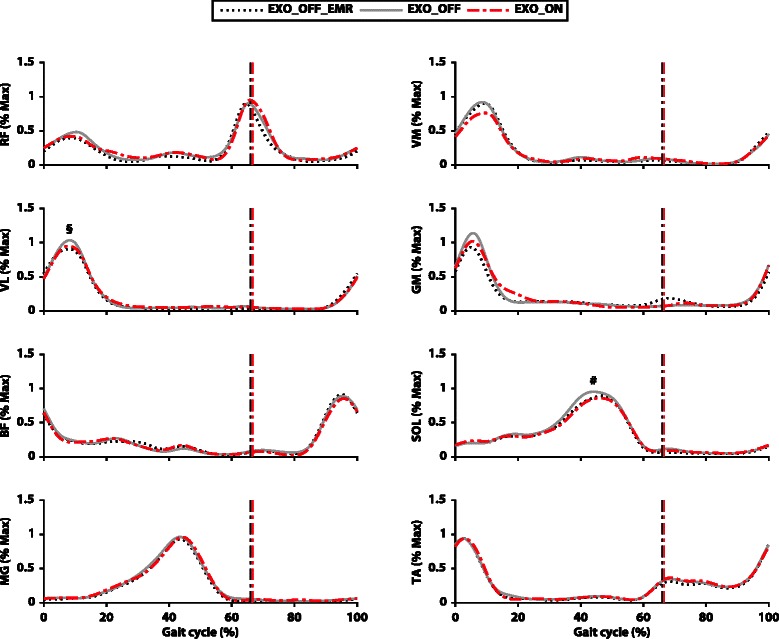
Muscle activation. Normalized EMG linear envelope as a percent of gait cycle (heel-strike to heel-strike) for the eight muscles examined. The curves represent the three different conditions: *EXO_OFF_EMR* (dashed black), *EXO_OFF* (solid grey) and *EXO_ON* (dashed red). The dotted vertical lines represent toe off of each testing condition. # indicates a significant difference (*p* < 0.05) with respect to the *EXO_OFF* condition, § indicates a significant difference (*p* < 0.05) between the *EXO_OFF_EMR* and the *EXO_OFF* condition

Significantly lower average muscle activation (4.7 ± 7.0 %) was reported in the vastus lateralis for the *EXO_OFF_EMR* condition with respect to the *EXO_OFF* condition (*p* = 0.005; ES = 0.47). Significantly lower average muscle activation (8.4 ± 9.8 %) was also reported in the soleus for the *EXO_ON* condition with respect to the *EXO_OFF* condition (*p* = 0.025; ES = 0.50). No main effect of muscle activation was reported for the six other lower limb muscles investigated. Muscle activations during the three conditions of testing are presented in Fig. 6 and in Additional file 1: Table S3.

### Spatio-temporal parameters

Stride length, stride frequency, duty factor and stance and swing times were not significantly different between the three testing conditions. A complete overview of these parameters is presented in Additional file 1: Table S4.

### Joints kinematics

**Fig. 7:**
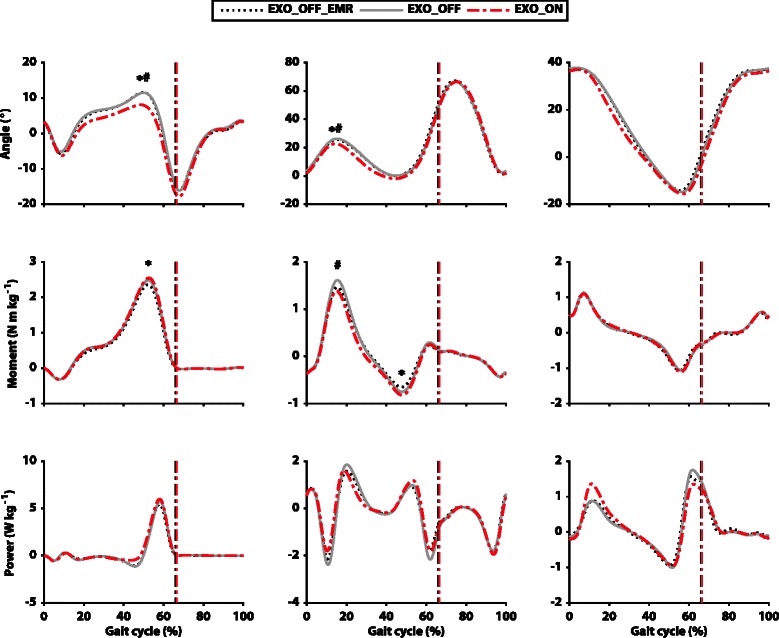
Joint kinematics and kinetics. Comparison of joint angles, moments and powers (top to bottom) for the three different conditions of testing across the gait cycle. The curves represent the three different conditions: *EXO_OFF_EMR* (dashed black), *EXO_OFF* (solid grey) and *EXO_ON* (dashed red). Ankle, knee and hip joints are displayed from left to right. Data are group means. The dotted vertical lines represent toe off. Positive joint angles represent flexion (dorsi-flexion at the ankle) and negative angles represent extension (plantarflexion at the ankle). Positive moments represent net extension joint moments (plantarflexion at ankle) and negative moments represent net flexion joint moments (dorsi-flexion at ankle). Positive powers represent instantaneous joint power generation and negative powers represent instantaneous joint power absorption. * Indicates significant difference (*p* < 0.05) with respect to the *EXO_OFF_EMR* condition, # indicates a significant difference (*p* < 0.05) with respect to the *EXO_OFF* condition

Significantly lower peak ankle dorsi-flexion angle was reported in the *EXO_ON* condition with respect to the *EXO_OFF_EMR* (*p* = 0.001; ES = 0.93) and the *EXO_OFF* (*p* = 0.002; ES = 0.81) conditions. Additionally, a significantly lower knee flexion angle peak was reported in the beginning of the stance phase in the *EXO_ON* condition with respect to the *EXO_OFF_EMR* (*p* = 0.012; ES = 0.36) and to the *EXO_OFF* (*p* = 0.015; ES = 0.40) conditions. No other significant differences were reported in kinematics (Fig. 7). A complete overview of joint kinematics data is presented in Additional file 1: Table S5.

### Joint kinetics

The only statistically significant differences in joint moments (Fig. 7), were a lower peak knee extension moment in the *EXO_ON* condition with respect to the *EXO_OFF* condition (*p* = 0.011; ES = 0.51) and a higher knee flexion moment peak in the *EXO_ON* condition with respect to the *EXO_OFF* condition (*p* = 0.001; ES = 0.30). Significantly higher ankle power generation was reported for the *EXO_ON* condition with respect to the *EXO_OFF_EMR* (*p* = 0.016; ES = 1.45) and to the *EXO_OFF* (*p* = 0.005; ES = 1.07) conditions. Additionally, significantly lower knee power absorption was reported in the *EXO_ON* condition with respect to the *EXO_OFF* condition (*p* = 0.003; ES = 0.87) and between the *EXO_OFF_EMR* condition and the *EXO_OFF* condition (*p* = 0.018; ES = 0.59). A complete overview of the joint kinetics data is presented in Additional file 1: Table S5.

### Biological joint work and power

Average peak forces generated by the exosuit across the seven participants were 272 ± 43 N of ankle plantarflexion, 204 ± 32 N of hip flexion, and 68 ± 24 N of hip extension. These external forces caused a significant reduction of total joint biological positive work (sum of hip, knee, and ankle) when comparing the *EXO_ON* condition with respect to the *EXO_OFF* condition (1.28 ± 0.26 J kg^−1^; *p* = 0.020; ES = 1.02) and to the *EXO_OFF_EMR* condition (1.22 ± 0.21 J kg^−1^; *p* = 0.007; ES = 0.86). Further, hip biological positive work was significantly reduced in the *EXO_ON* condition with respect to the *EXO_OFF* condition (*p* = 0.011; ES = 1.18) and to the *EXO_OFF_EMR* condition (*p* = 0.007; ES = 1.25), and ankle biological positive work was significantly reduced in the *EXO_ON* condition with respect to the *EXO_OFF_EMR* condition (*p* = 0.035; ES = 0.99). Total joint biological negative work was not significantly different between the three conditions; nevertheless, knee biological negative work was significantly reduced in the *EXO_ON* condition with respect to the *EXO_OFF* condition (*p* = 0.003; ES = 0.82).

Similarly, total joint biological positive power (sum of hip, knee, and ankle) was significantly reduced when comparing the *EXO_ON* (1.02 ± 0.10 W kg^−1^) with respect to the *EXO_OFF* condition (1.21 ± 0.19 W kg^−1^; *p* = 0.009; ES = 1.25) and to the *EXO_OFF_EMR* condition (1.16 ± 0.15 W kg^−1^; *p* = 0.007; ES = 1.10). Hip biological positive power was significantly reduced in the *EXO_ON* condition with respect to the *EXO_OFF* condition (*p* = 0.001; ES = 1.21) and to the *EXO_OFF_EMR* condition (*p* = 0.011; ES = 1.21), and ankle biological positive power was significantly reduced in the *EXO_ON* condition with respect to the *EXO_OFF* condition (*p* = 0.044; ES = 1.13). Total joint biological negative power was not significantly different between the three conditions. Knee biological negative power was significantly reduced in the *EXO_ON* condition with respect to the *EXO_OFF* condition (*p* = 0.004; ES = 1.20) and in the *EXO_OFF* condition with respect to the *EXO_OFF_EMR* condition (*p* = 0.020; ES = 0.85) (Fig. 5b).

## Discussion

The aim of this study was to evaluate the effects of an autonomous (fully portable) multi-joint soft exosuit on the metabolic cost of loaded walking. A net reduction in the metabolic cost was reported while walking with the exosuit compared to wearing the exoskeleton unpowered with effective mass removed. This finding (Fig. 5a) represents the first successful attempt to effectively reduce the metabolic burden experienced by load carriers with an untethered soft exosuit. In addition, this work also represents the first successful attempt to reduce the metabolic cost of walking with a multi-joint untethered wearable robot of any kind.

Recent work from two different research groups [30, 31] also demonstrated an augmentation of human walking by means of autonomous ankle exoskeletons. These two studies reported average reductions in metabolic cost of 11 ± 4 % and 7.2 ± 2.6 %, respectively, when the ankle joint was assisted during unloaded walking. An additional study [32] also reported an average reduction of 8 ± 3 % in metabolic cost during loaded walking using a device similar to [30]. Although the magnitude of metabolic reduction observed with the soft exosuit was similar to that reported by these recent studies, differences in our approach to augmenting human performance should be considered when comparing systems and outcomes. Indeed, [30, 32] actively provided assistance at the ankle joint only and delivered higher levels than we did with the soft exosuit at the ankle. This early embodiment of the soft exosuit and actuation units limited the maximum force that could be delivered to the wearer. However, by exploiting the legs being out of phase and timing synergy between hip flexion and ankle plantarflexion we developed an actuation scheme by which a single motor per leg could be used to assist multiple joints, enabling us to minimize system mass and distal mass in particular. Earlier work on multi-joint systems [9, 33, 34] did not report reductions in the metabolic cost of walking, likely due to high device mass.

As expected, the *EXO_ON* condition produced a larger reduction in metabolic cost when compared to the *EXO_OFF* condition than to the *EXO_OFF_EMR* condition. Although the relationship between muscle-tendon behavior and whole body energy consumption is complex [25], we hypothesized that the underlying physiological mechanisms regulating this interaction could be more pronounced in a *EXO_ON vs EXO_OFF* comparison and concealed, at least in part, by the additional load imposed by the system in the *EXO_OFF_EMR* condition.

Walking with the powered device did not alter participants’ spatio-temporal parameters, indicating that the exosuit’s assistive forces were not disruptive to participants’ freely-selected step frequencies and step lengths. Nevertheless, some alterations in participants’ kinematics and kinetics were present. Given the aforementioned finding of reduced metabolic cost during walking, we posit that these changes may have permitted the musculoskeletal system to operate more efficiently. Indeed, considering that ankle dorsiflexion and knee flexion have been shown to increase with an increase of load [5], our observed reduction of these two parameters in the *EXO_ON* condition suggests that the exosuit facilitated a return to gait patterns that resemble unloaded walking [5].

Walking with the soft exosuit reduced the total biological joint work produced by the lower limbs, and the most marked reduction in biological work production was at the hip joint. This reduction is of particular interest especially considering that the hip joint seems to have lower efficiency compared with the other lower limb joints [35]. This hypothesis is also supported by the fact that muscles crossing the hip have a reduced pennation angle and a longer fiber length than those crossing the ankle [36, 37]. This architectural difference, together with a shorter tendon, makes the elastic recoil of the hip muscle-tendon complex less effective compared to that of the ankle [35]. Consequently, work production at the hip during walking is more costly than at the ankle. Based on this rationale and our previous work [20], it seems possible that unloading the hip joint by means of our multi-joint exosuit could have resulted in a more effective energy saving strategy. Nevertheless, only future experimental studies decoupling the effect of the assistance for each joint could provide further elucidation on this point.

Although the main reduction in the biological work was reported at the hip, the knee and ankle may also have contributed to lowering the metabolic burden. Interestingly, the assistance provided by the soft exosuit reduced knee extension moments (Fig. 7) with an associated reduction in negative biological work. The knee mainly functions as a shock absorber during level ground walking [23], with the muscles spanning this joint performing mostly eccentric contractions [35]. This behavior is exaggerated during load carriage [3] and, although this type of contraction is more economical than an isometric or a concentric contraction [38], it is still associated with a metabolic cost. Therefore, the reduction in negative biological work at the knee may have contributed to a lower metabolic cost as well. Although the multi-articular textile load path does pass approximately through the center of the knee joint, it is possible that a small level of assistance was applied at the knee. However, given this, another hypothesis for the reduced work at the knee related to the assistance of the contralateral ankle joint can be presented, according to [39]. Contralateral ankle assistance is synchronized with the negative work generation at the knee during the gait cycle. An augmented push-off on the contralateral limb could have been beneficial to lower the load at the knee joint. To support this explanation, a higher ankle moment exhibited in the *EXO_ON* condition during push-off corresponded to a lower knee moment on the contralateral limb during weight acceptance (Fig. 7), similar to what has previously been reported by [39]. Moreover, a similar trend was present in positive ankle work during push-off and in the negative knee work on the contralateral limb during weight acceptance. Decreased production of biological ankle work was also reported in the *EXO_ON* condition with respect to the *EXO_OFF* condition. It can be assumed that the ankle joint also contributed to the metabolic reduction and this rationale can be supported by the reduced soleus activation in the *EXO_ON* condition with respect to the *EXO_OFF* condition. The reason this was not found when comparing the *EXO_ON* and *EXO_OFF_EMR* conditions may have been that the effect was masked by the wearer having to carry the increased mass of the device.

Surprisingly, despite the fact that biological work was significantly reduced, only small differences were found in muscle activation between conditions (Fig. 6). Reported variations of kinematics and kinetics may have been linked to changes in the functional properties of the muscles rather than simply being the result of reduced muscle activation. Although from a joint-level kinematics analysis it is impossible to decouple the individual behaviors of the tendon and muscle, these alterations might have been the result of muscle fascicles working in a more economical region of their force-length relationship. Previous work on an ankle exoskeleton designed for hopping [40], revealed adaptive changes in the fascicle length of the soleus associated with a reduction in metabolic cost. Nevertheless, at this stage this hypothesis remains speculative and only future work examining in vivo muscle properties could unravel the underlying specific muscle mechanism.

The observed variability in the reduction of metabolic cost between participants may be due to several contributing factors. The fixed external assistance across participants coupled with small variations in how the exosuit fit to different body sizes and shapes resulted in variations in the percentage of the delivered assistance relative to nominal biological joint torques at the hip and ankle. In addition, small variations in how the multi-articular load path crossed the knee may have resulted in small torques at the knee joint for some participants. Additionally, neuromuscular adaptation associated with the use of wearable robots remains an active research area [41], with little to no work done on loaded walking, and inter-individual adaptations may have contributed to the variation in our findings across subjects.

Based on the results of this study, we believe that there is potential for further enhancing the exosuit’s performance. First, it should be noted that the joint torques applied by the exosuit to the wearer were relatively low compared to the joint moments experienced by load carriers [3]. This was mainly due to the significant compliance in the textile component of the exosuit and its interface to the wearer. In recent work we have demonstrated that higher assistive forces can be delivered that both the ankle and hip with improvements to suit components and actuation units, demonstrating metabolic reductions up to 8.5 and 15 % respectively when assisting only hip extension and ankle plantarflexion with a multi-articular load path similar to that described in this work [42, 43]. These advances are driven by a more rigorous approach to soft exosuit component evaluation and characterization as we describe in [24, 44]. We believe that this will pave the way for future autonomous single and multi-joint soft exosuits that reduce the energy cost of both loaded and unloaded walking. Second, for the multi-joint exosuit presented in this paper, the timing for hip extension assistance was determined using sensor information from the ankle joint of the contralateral limb (as described in Additional file 1: Text S1). This limited the ability to precisely control how assistance was applied during hip extension, as demonstrated by the increased variability in the averaged torque profiles (Fig. 4) both for a given participant and across participants. To address this, we are developing new control approaches for the hip [42] and ankle [43] that provide more repeatable forces using inertial sensors located at each individual joint.

## Conclusions

Our results demonstrate that an autonomous soft exosuit can reduce the metabolic burden experienced by load carriers, possibly augmenting their overall gait performance. Although many basic fundamental research and development challenges remain in actuator development, textile innovation, sensing and control, this proof of concept study provides the first demonstration of a soft wearable robot to augment gait. This work also presented the first demonstration that an autonomous multi-joint wearable robot can achieve a metabolic reduction. Future studies will be necessary to explore the effects of assisting walking with a soft exosuit and explore if, for a given level of mechanical work, the most effective approach to providing assistance is by augmenting the function of a single joint or multiple joints. Moreover, the realization of devices that assist joints other than the ankle can increase the knowledge on the determinants of energy cost, potentially revealing different adaptive mechanisms of the musculoskeletal system. Finally, apart from assisting load carriers, we are exploring how the soft exosuit can be used as a platform to assist individuals with compromised ability to produce adequate forces during locomotion [45], paving the way for many translational opportunities of this technology across a range of different populations.

## Additional file

Additional file 1:
**Table S1.** Autonomous exoskeleton mass distribution, adapted from [32]. **Text S1.** Soft exosuit characterization (hip flexion), soft exosuit sensing and control system and assessment of *EXO_OFF_EMR* condition [46]. **Table S2.** Individual net metabolic power for the three different conditions of testing. **Table S3.** Percentage of EMG reduction with respect to the *EXO_OFF_EMR* condition. **Table S4.** Spatio-temporal parameters for the three different conditions of testing. **Table S5.** Joint kinetics and kinematics values for the three different conditions of testing. **Figure S1.** **a** Gyroscope profile employed to detect the heel strike event. T1 represents a peak in the signal that occurs consistently at 4 % in the gait cycle. **b** Force profile recorded by the load cell placed at the ankle. T2 represents the point at which the tension crosses 25 N, which triggers the actuation for the multiarticular load path. **c** Position of the cable actuating the multiarticular load path. **d** Position of the motor actuating the hip. T3 represents the beginning of the monoarticular actuation path of the contralateral limb. **e** Force profile recorded by the load cell placed at the contralateral hip.
