# Supplementary material for: A biologically-inspired multi-joint soft exosuit that can reduce the energy cost of loaded walking
Source: J Neuroeng Rehabil. 2016 May 12;13:43. doi: 10.1186/s12984-016-0150-9 (PMC4864923; doi:10.1186/s12984-016-0150-9)
Supplement: Additional file 1: — Table S1. Autonomous exoskeleton mass distribution, adapted from [32]. Text S1. Soft exosuit characterization (hip flexion), soft exosuit sensing and control system and assessment of EXO_OFF_EMR condition [46]. Table S2. Individual net metabolic power for the three different conditions of testing. Table S3. Percentage of EMG reduction with respect to the EXO_OFF_EMR condition. Table S4. Spatio-temporal parameters for the three different conditions of testing. Table S5. Joint kinetics and kinematics values for the three different conditions of testing. Figure S1. a Gyroscope profile employed to detect the heel strike event. T1 represents a peak in the signal that occurs consistently at 4 % in the gait cycle. b Force profile recorded by the load cell placed at the ankle. T2 represents the point at which the tension crosses 25 N, which triggers the actuation for the multiarticular load path. c Position of the cable actuating the multiarticular load path. d Position of the motor actuating the hip. T3 represents the beginning of the monoarticular actuation path of the contralateral limb. e Force profile recorded by the load cell placed at the contralateral hip. [file 12984_2016_150_MOESM1_ESM.pdf]

Additional files

Additional file 1:

Table S1. Autonomous exoskeleton mass distribution, adapted from [32]

Exoskeleton	Total mass (kg)	Mass foot (kg)	Mass shank (kg)	Mass thigh (kg)	Mass waist (kg)
Walsh et al. [9]	11.60	1.41	1.41	5.12	3.66
*Van Djik et al. [33]**	12.00	3.00	3.00	3.00	3.00
Mooney et al. [32]	3.60	0.19	1.93	0	1.48
*Collins et al. [31]**	0.91	0.37	0.54	0	0
Present study	6.60	0.04	0.62	0.45	5.49

* This exoskeleton is passive

Text S1.

Soft exosuit characterization (hip flexion)

The multiarticular load path transferred the bulk of the force up to the waist belt, but some force was transferred to the leg where it attached at the shin. To characterize the force distribution between these two points, the exosuit was instrumented with two additional load cells at the point where the leg straps (Figure 4B) connect to the waist belt. Three participants (age: 27.7±6.4 yr; height: 1.80±0.10 m; weight: 85.1±5.0 kg) walked for six minutes with the instrumented exosuit, and the peak force at the waist was compared to the peak force at the ankle for each step once the forces reached steady-state (Figure 4C). The ratio between peak force at the waist and peak force at the ankle was calculated for each participant using a minimum of 30 strides. This resulted in an average ratio of 76.95% across the three participants. Therefore, for simplicity, the force assisting the hip during flexion was assumed to be 75% of the force measured by the load cell at the ankle. To estimate the passive hip flexion moment generated by the exosuit, this force was multiplied by a constant moment arm of 10.7 cm, measured with a digital caliber on the three participants involved in this testing procedure. This hip flexion moment was subtracted from the joint moment in the biological work calculation estimate.

Soft exosuit sensing and control system

The controller was designed to effectively deliver forces that mimic the mechanics of the lower limbs. Therefore, the force profiles applied by the soft exosuit were chosen to mirror the biological joint torques [46], with the assumption that this would lower the load experienced by the joints.

To accomplish this task, two different sensors were utilized on each limb to detect key events during the gait cycle: a gyroscope at the heel, and a load cell at each location where a Bowden cable sheath attaches to the exosuit (at the hip and at the calf). The gyroscope was used to detect the contact of the foot with the ground (heel strike), which is defined as the beginning of the gait cycle. The two load cells were used to monitor the tension in the multiarticular and monoarticular load paths.

A force-based position control system was used to drive the actuators. This control imposed a predefined position trajectory to the motor acting on the Bowden cables in order to achieve a specific force profile. The cable position trajectory induced a force in the exosuit because the textile, the Bowden cables, and the human tissue underneath the exosuit are all compliant (Figure 2).

Although the functioning of the control system has been described in detail in [22], to enhance the clarity of the present manuscript, a brief description is presented below, using the right leg as an example. First, the heel strike event was defined based on the gyroscope signal that presented a constant peak at 4% in the gait cycle (T1) (Figure S1A). Following this event, the ankle motor returned to a pretension position after actuating the left leg. The multiarticular load path acting on the right leg then developed a passive force, with the motor holding a constant position, purely due to the kinematics changes of the wearer. Specifically, during this time in the gait cycle the ankle dorsiflexes and the hip extends. With these motions, the distances in the front of the hip and at the back of the ankle increase, thereby stretching the exosuit over the body and generating a tension force in it. The threshold to detect this tension was set to 25N and the corresponding event was defined as T2 (Figure S1B-C). The actuation for the multiarticular load path began immediately after T2, with the motor starting to actively pull the Bowden cable. The instantaneous walking speed of the wearer can be estimated from the previously defined points in the gait cycle (T1 and T2) thus allowing the calculation of a third point (T3) which corresponded to the beginning of the monoarticular actuation path of the contralateral limb. This point was set at the 46.5% of the gait cycle to start actuating the hip joint just before the heel strike (Figure S1D). The consequent hip actuation profile is then presented in Figure S1E.

The position profiles executed by the actuators were adjusted over time in order to maintain consistent forces over time and between users. In particular, the pretension positions and peak actuation positions were adjusted between successive steps in an iterative scheme to generate desired force profiles and pretension levels. After each step was completed, the true step period was known and used to compute when 36.5% in the gait cycle occurred (corresponding to time T2 in Figure S1B-C). The force at that time was compared to the desired value (25N), and the pretension position was adjusted by 1 mm in the appropriate direction for the subsequent step. Similarly, the pull amplitude was adjusted by 1 mm increments between steps in order to maintain a desired peak force for both the ankle and hip. When the system was initially powered on, this algorithm led to the actuation forces increasing slowly over time as the pretension positions and pull amplitudes were increased from small initial values. This controller was used because it

automatically corrects the position profile so that the desired forces are achieved independently of the way the exosuit is initially positioned for a particular wearer.

Assessment of *EXO_OFF_EMR* condition

We evaluated the *EXO_OFF_EMR* condition removing the equivalent weight of the multi-joint soft exosuit (6.6 kg) from the load in the backpack. This choice was taken for practical reasons during the testing protocol including the randomization of the conditions. Although it might be argued that removing the weight from the backpack (waist location) could not be equivalent to removing the whole exosuit because of the effect of mass distribution on metabolic cost [16], we believe that this effect was very small. To quantify it, we used the estimation for metabolic cost associated to weight distribution provided by [32]. With this calculation, we obtained that removing the whole exosuit would have accounted for 24.6 W, while removing the equivalent weight of the device, as in the present study, would have accounted for 24.4 W. This last estimate took into account also the effect of wearing a normal pair of pants (calculated for normal clothing of 0.6 kg of weight). The difference between the two estimates is likely to be negligible considering the average reduction of 35.2 W reported between the *EXO_ON* and the *EXO_OFF_EMR* condition, moreover, not having to remove the exosuit between conditions prevented changes in the backpack location that could have impacted on the variability. Based on this rationale we believe that it seems safe to conclude that our *EXO_OFF_EMR* condition could effectively be treated as a “no exosuit” condition.

Table S2. Individual net metabolic power for the three different conditions of testing

	Metabolic power (W)		
Participant ID	*EXO_OFF_EMR*	*EXO_OFF*	*EXO_ON*
1	427.5	470.6	422.1
2	451.2	503.3	392.5
3	433.4	460.5	382.5
4	566.5	586.3	546.9
5	487.2	529.4	477.8
6	537.2	557.6	491.2
7	513.8	585.9	457.3

Table S3. Percentage of EMG reduction with respect to the *EXO_OFF_EMR* condition

Muscle	*EXO_OFF*	*EXO_ON*
BF	0.0±14.6	-3.7±20.0
MG	8.6±11.4	8.7±8.5
GM	16.4±7.2	17.0±25.0
RF	16.5±22.9	18.2±20.1
SOL	8.4±9.8#	1.2±8.3
TA	4.7±11.0	7.0±15.4
VL	4.7±7.0 §	4.7±10.1
VM	1.6±19.7	-4.2±19.4

Data are means ± SD. § indicates a significant difference ($p < 0.05$) between the *EXO_OFF_EMR* and the *EXO_OFF* conditions, # indicates a significant difference ($p < 0.05$) between the *EXO_ON* and the *EXO_OFF* conditions.

Table S4. Spatio-temporal parameters for the three different conditions of testing

Condition	Stance time (s)	Swing time (s)	Duty factor	Stride freq. (Hz)	Stride length (m)
EXO_OFF_EMR	0.68±0.05	0.35±0.01	66.0±1.1	0.97±0.06	1.55±0.09
EXO_OFF	0.69±0.05	0.35±0.02	66.5±0.7	0.97±0.06	1.56±0.09
EXO_ON	0.70±0.05	0.34±0.03	67.2±2.2	0.97±0.06	1.56±0.09

Data are means ± SD.

Table S5. Joint kinetics and kinematics values for the three different conditions of testing

Variable	*EXO_OFF_EMR*	*EXO_OFF*	*EXO_ON*
Ankle angle (max)	12.0°±3.5°	11.7°±3.8°	8.7°±3.6° * #
Ankle angle (min)	-16.4°±2.8°	-16.5°±3.0°	-18.1°±2.7°
Knee angle (max)	66.6°±6.2°	67.4°±5.8°	66.7°±6.2°
Knee angle (min)	-1.2°±4.1°	1.8°±4.1°	-2.8°±3.8°
Hip angle (max)	38.8°±6.3°	39.1°±7.5°	38.2°±8.7°
Hip angle (min)	-14.4°±6.4°	-14.9°±7.1°	-15.8°±8.2°
Ankle moment (max) (Nm kg^{-1})	2.4±0.3	2.5±0.3	2.6±0.3*
Ankle moment (min) (Nm kg^{-1})	-0.3±0.1	-0.3±0.1	-0.3±0.1
Knee moment (max) (Nm kg^{-1})	1.5±0.4	1.6±0.5	1.4±0.4*
Knee moment (min) (Nm kg^{-1})	-0.7±0.2	-0.8±0.2	-0.8±0.2*
Hip moment (max) (Nm kg^{-1})	1.1±0.2	1.1±0.4	1.1±0.3
Hip moment (min) (Nm kg^{-1})	-1.1±0.2	-1.1±0.2	-1.1±0.1
Ankle positive power (W kg^{-1})	5.8±0.7	5.9±0.7	6.2±0.9
Ankle negative power (W kg^{-1})	-1.4±0.6	-1.3±0.5	-1.0±0.4
Knee positive power (W kg^{-1})	1.8±0.6	2.0±0.9	1.9±0.6
Knee negative power (W kg^{-1})	-2.5±0.7	-2.8±0.8	-2.3±0.4
Hip positive power (W kg^{-1})	1.8±0.2	1.8±0.2	1.8±0.3
Hip negative power (W kg^{-1})	-1.0±0.3	-1.1±0.4	-1.0±0.3

* Indicates significant difference ($p < 0.05$) with respect to the *EXO_OFF_EMR* condition, # indicates a significant difference ($p < 0.05$) with respect to the *EXO_OFF* condition. Data are means ±SD.

Figure S1.

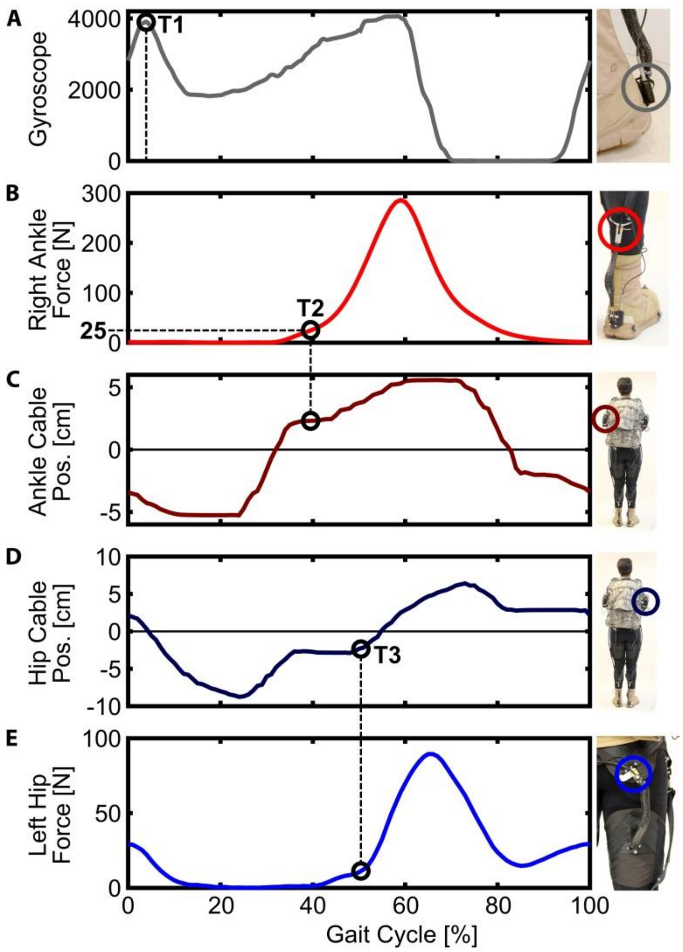

Fig. S1. **a** Gyroscope profile employed to detect the heel strike event. T1 represents a peak in the signal that occurs consistently at 4% in the gait cycle. **b** Force profile recorded by the load cell placed at the ankle. T2 represents the point at which the tension crosses 25 N, which triggers the actuation for the multiarticular load path. **c** Position of the cable actuating the multiarticular load path. **d** Position of the motor actuating the hip. T3 represents the beginning of the monoarticular actuation path of the contralateral limb. **e** Force profile recorded by the load cell placed at the contralateral hip
